# Er,Cr:YSGG Laser Therapy for Drug-Induced Gingival Overgrowth: A Report of Two Case Series

**DOI:** 10.3389/fsurg.2022.922649

**Published:** 2022-05-24

**Authors:** Yumei Liu, Qian Peng, Binjie Liu, Zhibin Wang, Qiong Cao

**Affiliations:** ^1^ Department of Radiology, Hunan Key Laboratory of Oral Health Research & Xiangya Stomatological Hospital & Xiangya School of Stomatology, Central South University, Changsha, China; ^2^ Department of Periodontology, Hunan Key Laboratory of Oral Health Research & Xiangya Stomatological Hospital & Xiangya School of Stomatology, Central South University, Changsha, China

**Keywords:** laser therapy, drug-induced gingival overgrowth (DIGO), surgery, aesthetics, oral health

## Abstract

**Background:**

Drug-induced gingival overgrowth is common but neglected in patients with systemic disease medications until it seriously affects the quality of life.

**Methods:**

Initial periodontal treatment, combined with water laser surgery, was performed sequentially in two cases.

**Results:**

The therapeutic effect was good, and there was no recurrence along with good oral hygiene.

**Conclusion:**

Water laser equipment surgery, as well as initial periodontal treatment, required that surgeons are trained specifically. A tool was devised for various oral diseases, and it was safer, more efficient and more comfortable than others.

## Introduction

Predisposing drugs for gingival enlargement mainly fall into three types: antiepileptic drugs (such as Phenytoin), immunosuppressive agents (such as Cyclosporin), and calcium channel antagonists (such as Nifedipine), while its severity can be modified by the degree of primary gingival inflammation and oral hygiene conditions (1). Gingival enlargement may lead to chewing and pronunciation difficulties regardless of oral facial aesthetics, and the major managements are initial periodontal treatment and surgical periodontal therapy. In the past few years, increasing studies have revealed the superiority of laser therapy over conventional surgical treatment, which manifested as less infection, better hemostatic effect, clearer surgical field, shorter operative time, less anesthetic dosage, and less postoperative discomfort with remarkable therapeutic effect (2). The water laser technique, other than having the above advantages, overcomes the deficiency of heat production caused by conventional laser techniques owing to its unique therapeutic mechanism, allowing its wide applicability in the treatment of diseases of oral soft and hard tissues. Here, we report the cases of two patients who underwent water laser-based gingivectomy for drug-induced gingival overgrowth (DIGO) following initial periodontal treatment. The patients suffered from slight bleeding and discomfort during the operation, with no postoperative pain or bleeding and showed rapid recovery. The gingiva of the patients gradually recovered to normal after 1 year of follow-up, and there were no signs of recurrence. Written informed consent was obtained from the patients for the publication of any potentially identifiable images or data included in this article.

## Case Description 1

### General Information

The first patient who was a male and 42 years old complained of 1 year of gingival overgrowth with bleeding from brushing at his first visit. Three years prior to this diagnosis, the patient underwent a kidney transplant in another hospital and took Tacrolimus and Felodipine after this. He had a history of kidney disease and hypertension but no allergies to medications or food.

### Examinations

#### Intraoral Examination (Figure 1)

The patient’s oral hygiene was in a poor condition, which manifested as a dental calculus (++), with a large amount of materia alba, swelling and tumor-like overgrowth of the gingiva, obtuse morphology, a nodular gingival enlargement of the anterior teeth covering more than 2/3 of the crown with a wide base, a barely noticeable pedicle, little mobility and a tough and substantial texture, PD: 4–6 mm, BI: 3–4.

**Figure 1:**
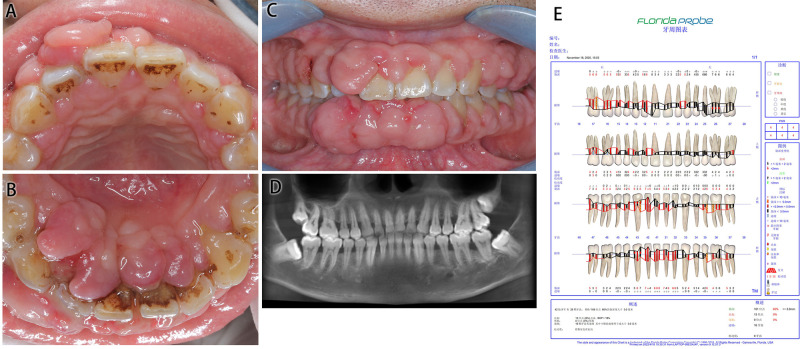
Medical information on Case 1 at first diagnosis (before treatment). (**A**) Maxillary view. (**B**) Mandibular view. (**C**) Frontal view images. (**D**) Radiological images. (**E**) Periodontal examination tables.

#### Extraoral Examination

The patient had facial symmetry, no obvious deformity or defects, normal mouth opening and shape, and no enlargements of bilateral cervical or submandibular lymph nodes.

#### Auxiliary Examination (Figure 1)

Cone beam computed tomography (CBCT) showed a slightly horizontal alveolar bone absorption of full teeth and a slightly vertical alveolar bone absorption of the bilateral posterior teeth. The periodontal examination tables show the presence of deep pockets around the periodontium.

#### Diagnosis

DIGO and chronic periodontitis were diagnosed.

#### Treatment Plan

Oral hygiene education; initial periodontal treatment; surgical periodontal therapy; periodontal maintenance therapy.

### Treatment Procedure

#### Initial Periodontal Treatment

Oral hygiene education was imparted for this patient. The patient was informed of the correct control methods for dental plaque and was advised to develop good oral hygiene habits. Complete supragingival and subgingival scaling and root planning were recommended successively (cleansing with hydrogen peroxide after treatment followed by local iodophenol application). Regular re-examination was required.

#### Surgical Periodontal Therapy

A total of 4–6 weeks after the periodontal therapy, the gingival overgrowth of the upper and lower anterior teeth reduced, while aesthetics, mastication, and oral hygiene maintenance were still affected. Er,Cr:YSGG laser was used for the removal of the enlarged gingiva at labial (palate) 13–23 and 33–43 based on the external oblique incision. The gingival morphology was trimmed and hemostasis was performed. Postoperative anti-inflammatory treatment and chlorhexidine gargle rinse were managed for 5 days.

#### Periodontal Maintenance Therapy (Figure 3)

One year after the operation, the patient’s oral hygiene was in good condition upon re-examination, with less plaque and pigmentation. The mouth gingiva was pinkish in color, with a tough texture and an improved gingival morphology, and there was no recurrence. The enlarged gingiva of the posterior teeth was not managed by periodontal surgery, but it greatly reduced after treatment.

## Case Description 2

### General Information

The second patient who was a female and 67 years old gradually developed gingival swelling with bleeding 6 years ago and visited our hospital for a recent difficulty in food intake. The patient suffered from diabetes, coronary heart disease and hypertension for more than 10 years. She had well-controlled blood glucose and pressure by daily oral administration of Metformin Hydrochloride tablets and Amlodipine Besylate tablets.

### Examination

#### Intraoral Examination (Figure 2)

This patient had poor oral hygiene, a large amount of plaque and materia alba and dental calculus (+++). The whole gingiva was bright red in color and manifested as spherical gingival enlargements covering more than 2/3 of the crown surface, with a tough texture and a tendency to bleed on touching. The upper and lower anterior teeth were spread, and teeth 31 and 41 were dislocated with 3° loosening.

**Figure 2:**
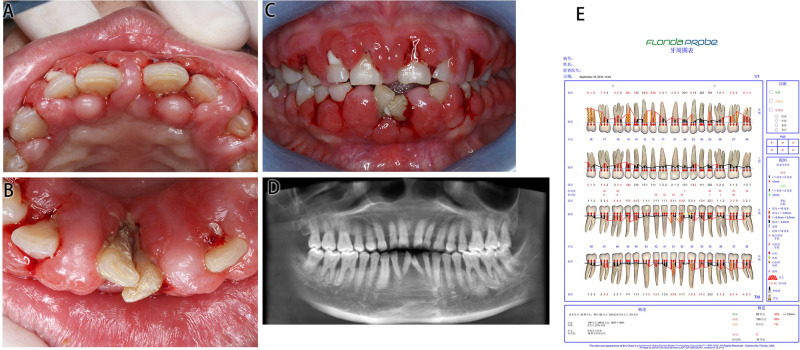
Medical information on Case 2 at first diagnosis (before treatment). (**A**) Maxillary view. (**B**) Mandibular view. (**C**) Frontal view images. (**D**) Radiological images. (**E**) Periodontal examination tables.

#### Auxiliary Examination (Figure 2)

CBCT presented an alveolar bone absorption of teeth 31 and 41 to the apical part, and an absorption of teeth 32 and 42 to the 1/3 apical part. Full mouth examination was referred to the periodontal specialist examination table.

#### Diagnosis

DIGO was diagnosed.

#### Treatment Plan

Oral hygiene education; initial periodontal treatment; surgical periodontal therapy; supportive periodontal therapy.

### Treatment Procedure

#### Initial Periodontal Treatment

Oral hygiene education was imparted. Complete supragingival and subgingival scaling and root planning were recommended to be performed consecutively. Inflammation was basically controlled in 4–6 weeks and regular re-examination was required.

#### Surgical Periodontal Therapy

Gingival swelling greatly subsided after initial treatment. The laser technique was used to generate a gingival physical appearance favorable for follow-up self-cleaning, by performing gingivectomy on 16–26 and 36–46 based on external oblique incision. The gingival morphology was trimmed and hemostasis was performed. The incision was protected by a periodontal pack. The patient was informed of postoperative precautions and asked to orally take antibiotics followed by chlorhexidine gargle rinse.

#### Supportive Periodontal Therapy (Figure 3)

In 1 week after the operation, the gingival swelling subsided significantly, and basically, a normal scallop-shaped gingiva was revealed.

**Figure 3:**
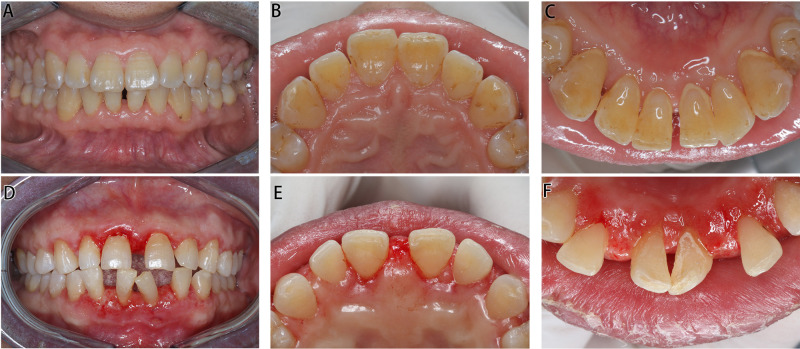
Intraoral images of Cases 1 and 2 post operation. (**A**–**C**) Images of Case 1 in 1 year post operation. (**D**–**E**) Images of Case 2 in 1 week post operation.

## Discussion

A transplant operation often requires the use of immunosuppressants such as Cyclosporine and Tacrolimus to reduce the rejection of recipients (3). Renal hypertension is prone to become a complication requiring a regular use of antihypertensive drugs, which is significantly associated with DIGO (4–6). Such disease is reported to occur at a probability range of 8%–70% (7), modified by a patient's age, drug dosage, duration of action, combination therapy, and other factors (8). Drugs of different types function behind diverse mechanisms. Phenytoin mainly causes gingival fibrosis, Cyclosporine is mainly associated with inflammatory responses with less effect on gingival fibrosis, while Nifedipine induces both fibrosis and inflammation, resulting in gingival overgrowth (9). Additionally, plaque microorganisms play an important role in the occurrence and development of DIGO (10, 11). Therefore, initial periodontal treatment is a necessity that can greatly control gingival enlargement-related inflammation by removing plaque and calculus, decreasing stimulation on periodontal tissue and reducing periodontal inflammation.

Predominantly, DIGO is managed by flap surgery, gingivectomy, and laser resection (12). Conventional gingivectomy involves a 45° oblique incision with a scalpel under anesthesia, which generates greater pain post operation (13) compared with flap surgery but preserves more aesthetic gingival morphology (14). However, the risks of anesthesia pain and accident, postoperative swelling and pain and infection cannot be negated. Besides, the surgical field of surgeons might be stained with the effects of massive bleeding during operation, and postoperative complications are prone to develop. Laser-guided gingival resection is emerging as an alternative with many advantages such as less infection, better hemostatic effect, clearer surgical field, shorter operative time, less anesthetic dosage, and less postoperative discomfort with remarkable therapeutic effect (2). Such a technique, on the one hand, makes up for the disadvantages in conventional gingivectomy, contributing to easier hemostasis, clearer surgical field and favorable therapeutic effect and prognosis. On the other hand, postoperative morphological recovery of gingiva is more aesthetic in nature owing to the procedure of oblique incision. Mavrogiannis et al. (13) compared the efficacy of traditional gingivectomy with laser resection on DIGO and postoperative recurrence, and they indicated that laser resection was superior in terms of hemostasis and the reduction of recurrence rates. Another compelling evidence by Campos et al. (15) also revealed that, in two recurrent cases of DIGO by laser treatment, only slight bleeding and discomfort occurred during operation with no postoperative pain, bleeding or recurrence signs in 1 year of follow-up. This indicated that laser treatment might be associated with increased therapeutic efficacy and favorable outcome of DIGO.

Currently, the main laser types available for gingivectomy are semiconductor diode laser, CO_2_ laser, Nd:YAG laser, Er:YAG laser, and Er,Cr:YSGG laser (16–19). Among these types, except the first one (semiconductor diode laser), the others are all capable of performing soft-tissue cutting and function to achieve hemostasis and sterilization (17, 20).

Er,Cr:YSGG laser therapy involves the release of a laser light of 2,780 mm in wavelength to activate water molecules and convert them into particles with high-speed kinetics, which enables the cutting function, and then causes the energy-released water to re-condense into normal water droplets. This allows the laser to perform its cutting function while protecting normal tissue and removing heat and debris from damaged tissue, which is in contrast to conventional surgery and the working of other laser types. The Er,Cr:YSGG laser during treatment can produce a kind of morphine-like electrical biological stimulation at the surgical site, which blocks nerve conduction and achieves analgesia (21). This can contribute to a reduction of pain during treatment, which is particularly suitable for those who are sensitive to pain, such as children and the elderly, and those incapable of tolerating pain. The patients in this report had poor pain tolerance due to DIGO induced by immunosuppressants and antihypertensive drugs after a kidney transplant. Er,Cr:YSGG laser resection was then managed for gingival enlargements, resulting in obvious discomfort during and after operation. The patients actively cooperated with the doctors and recovered well post operation. Soares et al. (22) applied the Er,Cr:YSGG laser to treat the case of gingival enlargement in a child, which showed remarkable results. Besides, favorable gingival healing was observed upon oral examination in 1 week and 3 months of follow-up visits. This is also in agreement with the views mentioned earlier. Current studies have identified that the Er,Cr:YSGG laser, apart from being used for the resection of an enlarged gingiva, is also used for soft tissue mass resection of gingiva and for the treatment of oral mucosal diseases and hard tissue diseases with favorable therapeutic effects.

### Patient Perspective

Due to its advantageous characteristics of efficient cutting function, less heat generation, less pain, active hemostasis, and coagulation, the Er,Cr:YSGG laser has been widely seen in oral and maxillofacial surgery, bone and soft tissue repair, and other oral surgeries (23). In addition, because of the safe, painless and comfortable treatment process, the fear of patients with regard to postoperative pain can be greatly alleviated, making it particularly suitable for children, the elderly and those incapable of tolerating pain. This also supports its promising application prospect. Although Er,Cr:YSGG laser equipment, as well as treatment cost, is huge, and surgeons are required to be trained specifically in its use, it is a tool that is safer, more efficient and more comfortable than others for the treatment of various oral diseases.

## Data Availability

The raw data supporting the conclusions of this article will be made available by the authors, without undue reservation.
